# Botulinum Toxin in the Treatment of Hair and Scalp Disorders: Current Evidence and Clinical Applications

**DOI:** 10.3390/toxins17040163

**Published:** 2025-03-25

**Authors:** Sofia M. Perez, Sarah A. AlSalman, Betty Nguyen, Antonella Tosti

**Affiliations:** 1 Dr Philip Frost Department of Dermatology and Cutaneous Surgery, University of Miami Miller School of Medicine, Miami, FL 33136, USA; 2 Department of Dermatology, King Abdulaziz Medical City, Riyadh 22490, Saudi Arabia

**Keywords:** alopecia, hair loss, telogen effluvium, folliculitis decalvans, dissecting folliculitis, seborrheic dermatitis, hyperseborrhea, cutaneous piloleiomyomas, linear scleroderma, morphea, scalp pain, scalp dysthesia, trichodynia, hyperhidrosis, botox, neuromodulator, neurotoxin, onabotulinumtoxin, incobotulinumtoxin, abobotulinumtoxin, rimabotulinumtoxin, prabotulinumtoxin

## Abstract

Botulinum toxin (BoNT) is well-recognized throughout dermatology for its cosmetic indications and growing therapeutic value. Recent studies have trialed BoNT in the treatment of hair and scalp disorders, many of which lack long-term effective treatments and significantly impact quality of life. In this review, we summarize the current clinical literature on this topic to comprehensively evaluate the efficacy, safety, and clinical value of BoNT in treating hair and scalp conditions. A literature search on PubMed/MEDLINE and Scopus identified 40 articles reporting the use of 25–200 units of BoNT-A or B in 689 patients with hair loss (79.5%), scalp seborrheic dermatitis/hyperseborrhea (10%), craniofacial hyperhidrosis (9%), folliculitis decalvans/dissecting folliculitis (0.86%), scalp pain (0.43%), or linear scleroderma (0.29%). Most studies on BoNT therapy for androgenetic alopecia (AGA) reported mild or non-significant hair growth; however, considerable variability in outcome measures complicates the ability to draw definitive conclusions or justify the use of BoNT over established AGA therapies. BoNT-A and B showed consistent efficacy in treating craniofacial hyperhidrosis with minimal side effects. Additional scalp conditions may benefit from BoNT therapy, but the evidence is limited, and larger, controlled studies are needed to better understand BoNT’s clinical value in these conditions.

## 1. Introduction

Botulinum toxin (BoNT), derived from *Clostridium botulinum*, is well-known for its cosmetic use and therapeutic benefits in various neurological and dermatologic disorders. While numerous subtypes of *C. botulinum* toxins exist, denoted A through G, only subtypes A and B are available for clinical use [1]. Clinical formulations of BoNT-A and B including onabotulinumtoxin A, incobotulinumtoxin A, abobotulinumtoxin A, rimabotulinumtoxin B, and prabotulinumtoxin A differ slightly in their toxin concentration and are each approved by the United States Food and Drug Association (FDA) for various dermatologic indications [2]. The wide range of therapeutic benefits, including pain relief and anti-inflammatory effects, has driven the increased off-label use of BoNT in various dermatologic conditions including eczema, rosacea, idiopathic pruritus, keloidal scars, and hair disorders [2,3].

Hair disorders encompass a number of pathologies that differ in underlying cause and clinical manifestations. Androgenetic alopecia (AGA), also known as male or female pattern hair loss, is the most common hair loss disorder, affecting over 50% of men and 25% of women over the age of 50 [4]. Classically, AGA manifests as gradual thinning and eventual loss of terminal hairs across androgen-dependent regions of the scalp [4]. In genetically pre-disposed individuals, higher production of dihydrotestosterone (DHT) and consequent overactivation of hair follicle androgen receptors leads to follicular miniaturization through a progressive shortening of the anagen (growth) phase [5,6]. Standard treatments, such as minoxidil and finasteride, are aimed at prolonging the anagen phase through increased follicular blood flow and oxygen supply [7,8,9] and at decreasing DHT production by inhibiting 5-alpha-reductase, respectively [10,11]. Preliminary in vitro and animal studies have demonstrated the mechanistic potential of BoNT to similarly promote hair growth in AGA by increasing scalp blood flow, and oxygenation, and inhibiting DHT-induced miniaturization through antagonizing key cytokines like TGF-b1 [12,13,14]. However, its efficacy in clinical studies may not reflect these promising preclinical results.

While much of the recent literature has focused on AGA, BoNT’s role in other hair disorders including alopecia areata (AA), telogen effluvium (TE) and cephalalgia alopecia has also been explored [15,16]. A recent study of BoNT-A injections in mice models of wound healing demonstrated potent anti-inflammatory effects including decreased levels of pro-inflammatory M1 macrophages through BoNT-A inhibition of the JAK2/STAT1 pathway [17]; a signaling pathway targeted by novel biologic therapies in the treatment of AA [18,19]. BoNT’s potent inhibition of neurotransmitters like acetylcholine, substance P, and calcitonin gene-related peptide (CGRP) [20] may hypothetically improve the symptomatic treatment of hair and scalp conditions associated with scalp pain, dysthesia, or trichodynia [21,22].

Craniofacial hyperhidrosis is characterized by excessive sympathetic stimulation of eccrine gland sweat production localized to the face, head, or scalp [23]. OnabotulinumtoxinA, which is FDA-approved for the treatment of axillary hyperhidrosis, has preliminarily demonstrated promising therapeutic efficacy in craniofacial hyperhidrosis; however, it is not yet an FDA-approved clinical indication [24]. Notably, increased scalp sweating in a number of patients with inflammatory hair loss disorders suggests a potential association between increased sweating and scalp inflammation, where BoNT may additionally provide symptomatic benefit [25].

Recently, several studies have trialed the use BoNT in the management of hair and scalp conditions. In this article, we aim to comprehensively review the current clinical literature on this topic to present a critical evaluation on the efficacy, safety, cost-effectiveness, and clinical value of BoNT in the treatment of hair and scalp conditions.

## 2. Methods

We searched PubMed/MEDLINE and Scopus for articles published before November 2024 using the search terms “hair” OR “scalp” OR “alopecia” and their associated MESH terms, along with BoNT-related keywords, including all clinical formulations and brand names.

**Figure 1:**
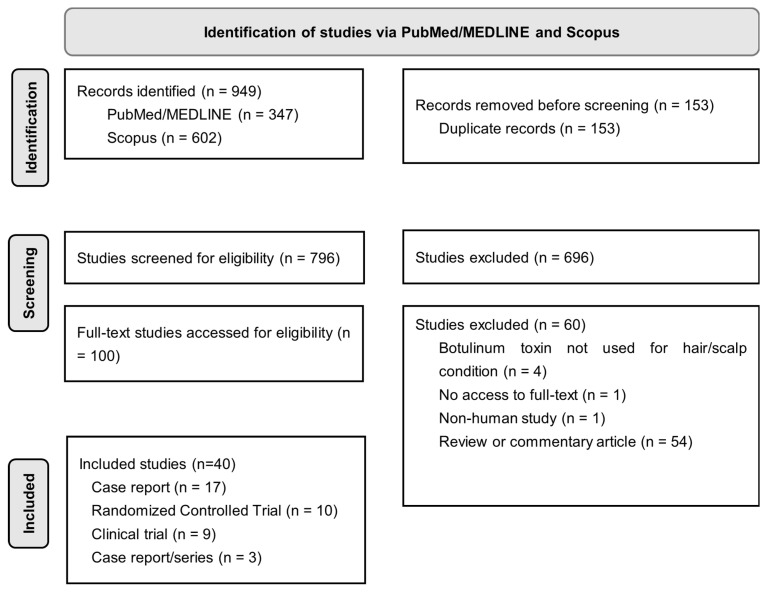
Flowchart of study identification according to the Preferred Reporting Items for Systematic Reviews and Meta-Analyses (PRISMA) guidelines.

This review was registered on PROSPERO (ID: CRD42025641897). All resulting articles were uploaded into Covidence web-based software (Covidence systematic review software, Veritas Health Innovation, Melbourne, Australia, access date: 29 October 2024. https://www.covidence.org/) for removal of duplicates, screening, and full-text evaluation. Screening and review of articles was conducted according to Preferred Reporting Items for Systematic Reviews and Meta-Analyses (PRISMA) guidelines (Figure 1). Two researchers independently reviewed titles and abstracts to remove unrelated articles. Only articles that described patients with a diagnosed hair or scalp condition who received intradermal or intramuscular injections of BoNT as primary or adjuvant therapy were included. Articles were excluded if they were non-human studies, lacked full text (i.e., abstract only), or involved BoNT use for other clinical indications. Full-text review was conducted by two independent researchers and discrepancies were resolved by discussion and mutual agreement.

## 3. Results

### 3.1. Study Characteristics

Of the 949 articles yielded from our search, 40 articles (17 case reports, 10 randomized controlled trials, 9 clinical trials, 3 case reports/series, and 1 prospective cohort study) met inclusion criteria (Figure 1).

Included studies described 689 patients treated with 25–200 units (U) of BoNT-A or B for the following hair and scalp disorders: hair loss (79.5%, 548/698) (androgenetic alopecia [74.2%, 511/698], telogen effluvium [3.5%, 24/698], cephalalgia alopecia [0.43%, 3/698], alopecia areata [1%, 7/698], trichotillomania [0.14%, 1/698], filler-induced alopecia [0.14%, 1/698], radiotherapy-induced alopecia [0.14%, 1/698]), seborrheic dermatitis/hyperseborrhea (10%, 69/698), craniofacial hyperhidrosis (9%, 62/698), folliculitis decalvans/dissecting folliculitis (0.86%, 6/698), scalp pain (0.43%, 3/698), or linear scleroderma (0.29%, 2/698).

Formulations of BoNT used included onabotulinumtoxin A (Botox), Chinese botulinum toxin type A (Hengli), abobotulinumtoxin A (Dysport), prabotulinumtoxin A (Nabota), and rimabotulinumtoxin B (NeuroBloc).

### 3.2. Botulinum Toxin Efficacy

The efficacy of BoNT in the treatment of hair loss, craniofacial hyperhidrosis, and other scalp disorders are presented in Table 1, Table 2 and Table 3, respectively. Individual study details including BoNT dosage and injection protocol are also described.

### 3.3. Botulinum Toxin Safety

In all 40 included studies, reported adverse events due to BoNT-A injections included scalp irritation (12.5%), headache (1.5–31%), nausea (1.5%), and injection site reactions like pain, erythema, and edema in 3.1–4.7% of patients [33,35,59]. Reported adverse events due to BoNT-B injections included forehead stiffness (18–66.7%), eyebrow drooping (18%), headache (3%), nausea (33%), increased sweating (11–33%), dry mouth (3–33%), and injection site reactions like skin dryness and bruising (3–5%) [46,47].

In one randomized controlled trial of 29 patients with AGA, one patient reportedly developed a patch of AA on the occipital scalp 3 weeks following BoNT injections [31]. Treatment with topical corticosteroids resulted in prompt hair regrowth. The possibility that the patch was in reality caused by pressure alopecia due to excessive volume injection cannot be excluded.

## 4. Discussion

### 4.1. Hair Loss

**Figure 2:**
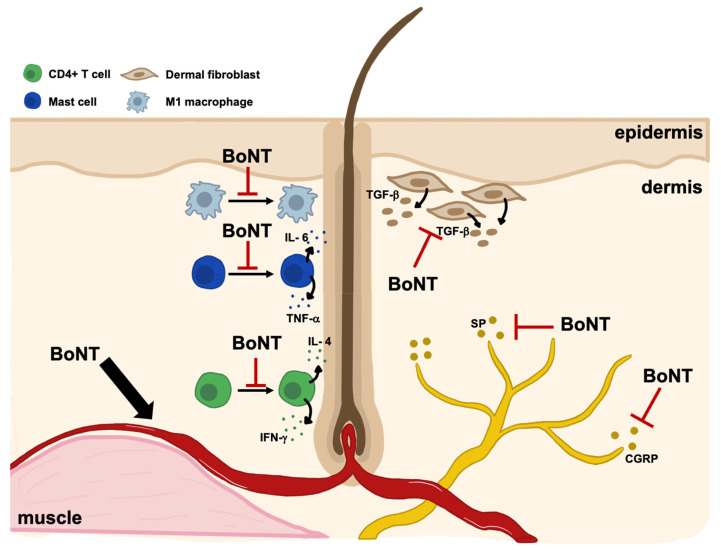
Hypothesized mechanisms of botulinum toxin’s therapeutic effects in hair disease. Left: BoNT increases vascular supply and oxygenation to hair follicles by relaxing scalp musculature and reducing pressure on local vasculature [12,65]. Middle: BoNT decreases perifollicular infiltration of CD4+ T cells [66], mast cells [67], and M1 macrophages [17]—all classically found in the lymphocytic infiltrate of alopecia areata [68] and, to a lesser extent, androgenetic alopecia [69,70]. BoNT also antagonizes dermal fibroblast secretion of the pro-fibrotic and pro-inflammatory cytokine TGF-b [12,71]. Right: BoNT inhibits the release of SP and CGRP from dermal nerve fibers [20]. When overproduced, SP and CGRP may contribute to physical symptoms of scalp disease (i.e., scalp burning, itching, pain) or stress-induced hair loss [72,73,74]. BoNT: botulinum toxin, TNF: tumor necrosis factor, IL: interleukin, IFN: interferon, TGF: transforming growth factor, SP: substance P, CGRP: calcitonin gene-related peptide.

The human hair cycle is a complex and tightly regulated process that oscillates between an active growth phase (anagen), apoptosis-driven regression phase (catagen), and resting (telogen) phase [63]. This cycle is highly influenced by a number of endogenous agents (e.g., oxygen, nutrients, hormones, neurotransmitters, cytokines) and exogenous stimuli (e.g., drug use, stress, and hair care practices) that if disrupted, can lead to a number of hair loss pathologies [63,64]. Given the complex nature of the hair growth cycle and hair disease pathogenesis, treatments are commonly aimed at a variety of regulatory components including follicular blood supply (minoxidil), hormonal influence (finasteride, dutasteride), inflammatory cytokine signaling (Janus kinase inhibitors, topical corticosteroids), and endogenous growth factors (platelet-rich plasma). Similarly, BoNT is also hypothesized to exhibit therapeutic efficacy in hair disease via its multifactorial influence on blood flow, local inflammation, and neurotransmitter activity (Figure 2) [12,65].

As a potent inhibitor of acetylcholine release, BoNT prevents muscle contraction which may reduce pressure on surrounding vasculature, increasing local blood flow [65,75]. BoNT’s ability to increase cutaneous blood supply [14,76,77] is hypothesized to promote hair growth by prolonging the anagen growth phase and by increasing follicular oxygen supply [12,65]. Both of these therapeutic functions are linked to underlying pathological processes in AGA: BoNT’s prolongation of the anagen phase may combat AGA-associated anagen shortening and BoNT’s increased hair follicle blood flow may improve scalp hypoxia in AGA [78,79,80].

Successful attenuation of cutaneous inflammation with BoNT has been demonstrated in animal studies [17,66,67] and supported with clinical evidence in the treatment of inflammatory skin conditions [81,82,83,84]. In mouse models of psoriasis, a one-time BoNT-A injection significantly decreased cutaneous infiltration of CD4^+^ lymphocytes [66]. In another study, BoNT-treated mice models of wound healing demonstrated inhibition of pro-inflammatory JAK2/STAT1 signaling and reduction in pro-inflammatory M1 macrophages [17]. These therapeutic functions are linked to underlying pathological processes in AGA and AA: BoNT’s suppression of CD4^+^ lymphocytes may antagonize their inflammatory activity in AGA [69,70] and BoNT’s inhibition of JAK/STAT signaling may block hair follicle immune privilege collapse in AA [68].

TGF-b is another pro-inflammatory cytokine that is influenced by BoNT-A. Dermal fibroblasts are a major source of TGF-b production which both perpetuates inflammation [85] and induces perifollicular fibrosis [86]. In vitro and in vivo studies have demonstrated an inhibitory effect of BoNT on both fibroblast proliferation [71] and expression of TGF-b [12]. These therapeutic functions are linked to underlying pathological processes in AGA and scarring alopecias: BoNT’s inhibition of fibroblast proliferation and TGF-b production may antagonize the DHT-induced upregulation of TGF-b and its subsequent pro-inflammatory signaling, fibrosis, and hair follicle miniaturization in AGA [86,87]. In scarring alopecia, BoNT’s inhibition of dermal fibroblast proliferation may attenuate peri- and intrafollicular fibrosis [88].

Hair follicles are richly innervated by both sensory and autonomic nerves [89,90]. While precise details of nerve-hair follicle interactions remain relatively unclear, hair loss pathologies associated with neurologic disorders or psychological stress [90] demonstrate the important influence of neurotransmitters and neuropeptides on the hair cycle [91,92]. Cephalalgia alopecia is a rare neurological condition characterized by recurrent burning or stabbing pain in a region of the scalp with colocalized hair loss [38]. Etiology likely involves repetitive activation of nociceptive C fibers resulting in headache/scalp pain and a large release of substance P (SP) and calcitonin gene-related peptide (CGRP) that triggers a perifollicular inflammatory response [72] and upsets the balance of trophic SP and CGRP levels necessary for normal hair growth [73,74]. BoNT’s ability to inhibit the release of SP and CGRP from nerve fiber terminals [20] therefore presents ideal therapeutic potential for both hair regrowth and temporary pain alleviation in cephalalgia alopecia [38,39].

Despite these encouraging mechanistic hypotheses from pre-clinical studies, the clinical role of BoNT in hair disorders is not as promising.

Clinical studies reporting BoNT in the management of AGA present variable evidence regarding its efficacy. While three studies reported a significant increase in hair growth after 2–5 sessions of BoNT-A [12,26,33], multiple randomized controlled trials demonstrated no significant difference from baseline [12,26,31,32,33,34,43], or when BoNT was used in combination with topical minoxidil and oral finasteride [32]. The reason for this discrepancy may be due to differences in BoNT dosage and evaluation methods used (Table 1). Studies demonstrating statistical significance tended to use higher total doses of BoNT (100–180 U) distributed across many regions of the scalp and over 2–6 sessions within 24–48 weeks, noting statistical significance only after a minimum of 2 sessions [12,26,33]. However, a recent randomized controlled trial similarly used 50 U of BoNT-A over 2 sessions separated by 12 weeks and reported no significant difference in hair growth from baseline or compared to placebo [43]. Notably, this trial used only a small 1 cm^2^ region of the scalp to inject BoNT and assess change in hair growth. Previous studies have employed less reliable methods to document BoNT efficacy including manual hair counts, global photographic assessment, and patient or physician-reported improvement [12,30,33,41,42].

In comparison to standard AGA therapies including topical minoxidil and oral finasteride, which have both demonstrated significant clinical efficacy in a number of randomized controlled trials [93,94,95,96]. BoNT seems to be a relatively low-evidence option for AGA management. One additional concern for clinical use of BoNT involves its significantly high cost ($600/100-unit vial, BOTOX^®^) compared to standard AGA treatment options (3-month supply 5% topical minoxidil: $47.99, 90-day supply 1 mg oral finasteride: $28.36) [97]. Recent literature has brought into question this practical aspect of BoNT use in AGA and calls for further evidence-based data in support of its superior efficacy in order to justify this cost discrepancy [97]. One way to optimize cost-effectiveness may be to use BoNT in combination with other “skin boosters” like platelet-rich plasma (PRP). Skin boosters are substances that stimulate collagen production and overall skin rejuvenation when injected into the dermis [98]. Many skin boosters including hyaluronic acid, poly-L-lactic acid, and PRP combat oxidative stress and skin aging, which may contribute to AGA [99]. However, PRP is currently the only skin booster with robust literature to confirm its evidence-based efficacy in AGA therapy [100,101] and future studies are needed to assess its synergistic effect when used with BoNT.

### 4.2. Craniofacial Hyperhidrosis

Similar to its mechanism in axillary hyperhidrosis, BoNT inhibits the release of acetylcholine, effectively blocking the excessive sympathetic stimulation of eccrine sweat glands [23]. The therapeutic efficacy of BoNT in treating craniofacial hyperhidrosis is consistently demonstrated with objective assessments (gravimetric analysis of sweat rate) and subjective measurements (DLQI, patient-reported improvement) throughout the 8 included studies [46,47,48,49,50,51,52,53]. Although no head-to-head comparison studies were found, the efficacy of BoNT-A seems to be relatively equitable to that of BoNT-B. Main clinical concerns include potential functional or cosmetic defects due to BoNT-induced facial muscle weakness [23]; however, eyebrow drooping was only reported in one of our included studies involving 7/38 (18%) of patients [47]. Forehead stiffness was the most common adverse event, reported by up to 67% of patients in one randomized controlled trial [46].

### 4.3. Other Scalp Conditions

While the use of BoNT in additional scalp conditions has been documented, evidence supporting its efficacy is variable and based solely on case reports and small observational studies. In contrast to the increasing evidence in support of BoNT for facial sebum production in acne treatment [102], we identified only two trials reporting BoNT for scalp sebum production [55,59]. Both studies reported a reduction in scalp sebum production with 50–100 U BoNT-A; however, this reduction was only statistically significant in one study [59].

The pathogenesis of folliculitis decalvans is still unclear and current treatment strategies including systemic antibiotics and steroids demonstrate limited efficacy in improving the disease severity or associated symptoms [103]. In the two studies we identified describing BoNT therapy for this condition, the therapeutic response was promising and resulted in decreased scalp secretions and even hair regrowth in 4 patients [56]. Notably, all patients were taking oral antibiotics during or shortly before initiation of BoNT therapy. A mechanistic explanation for this potential efficacy is lacking; however, it may involve BoNT’s inhibitory effect on fibroblasts and perifollicular fibrosis [88], or BoNT’s anti-inflammatory properties, since an excessive inflammatory response to scalp bacteria likely propagates this condition [104].

Scalp pain, dysthesia, or trichodynia can be caused by multiple hair, scalp, or systemic conditions [105]. Studies reporting BoNT for symptomatic management of scalp pain demonstrate promising efficacy, although effects are temporary and return of symptoms reportedly occurs within 3–4 months post-treatment [22,54,61]. Evidence is limited to case reports of scalp pain in the setting of cutaneous piloleiomyomas and idiopathic trichodynia or scalp dysthesia. It is unknown whether these therapeutic results are applicable to scalp pain of other etiologies.

Localized scleroderma involving the scalp may manifest as an atrophic, linear patch of alopecia termed linear scleroderma en coup de sabre [106]. Although rare, en coup de sabre morphea can be associated with neurologic symptoms, including migraines and trigeminal neuralgia [107]—both symptoms that have previously demonstrated a positive response to BoNT treatment [108,109]. BoNT’s ability to enhance blood flow may further contribute to its efficacy in this condition since one proposed mechanism behind en coup de sabre morphea involves aberrant vasoconstriction-induced skin and muscle atrophy [107]. In our study, we present two cases where BoNT provided cosmetic improvement to the frontotemporal patches of morphea [60,62]. While this treatment strategy is significantly limited by minimal evidence, it does provide a possibly favorable alternative to other cosmetic improvement options in en coup de sabre which typically involves invasive procedures like reconstructive surgery or fat grafting [107].

## 5. Conclusions

Many promising mechanistic hypotheses for the role of BoNT in the treatment of hair and scalp conditions have been presented in the literature; however, a thorough assessment of its clinical use is essential if BoNT hopes to be introduced as a standard treatment option for these conditions. In this review, we present the current clinical evidence of BoNT use in hair disorders, craniofacial hyperhidrosis, scalp hyperseborrhea, folliculitis decalvans, scalp pain, and linear scleroderma.

Evidence of BoNT in AGA has been presented in 6 clinical trials, 7 randomized controlled trials (RCTs), and 1 prospective cohort study with conflicting results and significant heterogeneity among BoNT dosage and evaluation methods. Based on three recent RCTs with robust sample sizes, BoNT-A does not significantly increase hair growth in AGA and is not a cost-effective treatment option. Future studies should include multicenter RCTs using established evaluation methods to assess short and long-term outcomes of BoNT in AGA, either alone or in combination with topical/oral minoxidil, anti-androgens, or skin boosters like PRP.

Evidence of BoNT in craniofacial hyperhidrosis has been presented in 2 clinical trials, 1 RCT, 4 case reports, and 2 case series demonstrating notable efficacy and little to no adverse events. Based on the 2 clinical trials and RCT, BoNT significantly decreases sweating of the face and scalp and should be considered an effective treatment for craniofacial hyperhidrosis. Future studies should include multicenter RCTs to evaluate the long-term efficacy and optimal dosing and injection frequency of BoNT treatment.

Evidence of BoNT in all other hair/scalp conditions (i.e., folliculitis decalvans, scalp pain, linear scleroderma) is weakly supported by evidence-based data and results should be interpreted with caution given their preliminary nature and need for further validation. Future studies should include larger cohorts and objective assessment of symptomatic and physical improvements with BoNT alone or in combination with other therapies.

## Figures and Tables

**Table 1:** Studies describing patients who received BoNT injections in the scalp for the treatment of hair loss conditions, organized in order of descending injection dose.

Title, Author and Date	Study Type	# of Patients	Hair/Scalp Condition	BoNT Type (Formulation)	Dose	Results	Adverse Events (AEs)
180 U BoNT							
The effect of intradermal botulinum toxin on androgenetic alopecia and its possible mechanism Shon et al., 2020 [12]	Clinical trial	18	AGA	Prabotulinumtoxin A (Nabota^®^, Daewoong Pharmaceutical Co., Hwaseong, Gyeonggi Province, South Korea)	Total dose: 180 U, 20 intradermal injection sites on entire scalp	Hair density assessment Hair density (hairs/cm^2^) Baseline: 129.61 12 weeks: 129.11 24 weeks:136.22 *p* = 0.803, comparison between baseline and week 12 *p* = 0.012, comparison between baseline and week 24	No AEs reported
150 U BoNT							
Treatment of male pattern baldness with botulinum toxin: a pilot study Freund et al., 2010 [26]	Clinical trial	50	AGA	OnabotulinumtoxinA (BOTOX^®^, Allergan, Irvine, CA, USA)	Total dose: 150 U, 30 intramuscular injection sites on entire scalp	Hair density assessment Hair density (hairs/cm^2^) Baseline: 234 48 weeks: 276 *p* = 0.001	No AEs reported
Treatment effects of intradermal botulinum toxin type A injection on alopecia areata Cho et al., 2010 [27]	Clinical trial	7	AA	OnabotulinumtoxinA (BOTOX^®^, Allergan, Irvine, CA, USA)	Total dose: 150 U, three intradermal injections in lesional AA patches	Subjective improvement assessment Patient 1: Spontaneous hair regrowth after 2 months Patient 2: No response Patient 3: No response Patient 4: No response Patient 5: No response Patient 6: No response Patient 7: Aggravation and progression of disease severity	No AEs reported
Recent modalities in treatment of telogen effluvium: Comparative study Khattab 2022 [28]	Randomized clinical trial	12	TE	BoNT-A (REFINEX^®^, KC Pharmaceuticals, Pomona, CA, USA)	Total dose: 150 U, intramuscular injection sites on entire scalp	Statistically significant improvement in all hair parameters (increase in total hair count, terminal hairs, and multiple hair follicular units; decrease in vellus hairs and single follicular units) at 6 months post-treatment	No AEs reported
Radiation-induced Alopecia Treated with Botulinum Toxin Type A Injection Hyun et al., 2014 [29]	Case report	1	Radiation-induced alopecia	Botulax (Hugel Pharma, Seoul, Korea)	Total dose: 150 U, 30 intradermal injection sites in alopecic region	Subjective clinical assessment 3 months post-treatment: Sparse vellus hairs observed 12 months post-treatment: Improved hair density and thickness, some regrowth on the frontal scalp	No AEs reported
A Pilot Study to Evaluate Effectiveness of Botulinum Toxin in Treatment of Androgenetic Alopecia in Males Singh et al., 2017 [30]	Clinical trial	10	AGA	BoNT-A (unspecified)	Total dose: 150 U, 30 intramuscular injection sites on entire scalp	Clinical improvement assessment Physician-rated assessment Excellent: 8/10 (80%) Fair: 1/10 (10%) Poor: 1/10 (10%)	No AEs reported
100 U BoNT							
Intradermal Injection in Balding Region Versus Intramuscular Injection in Surrounding Muscles: A Split-Scalp, Randomized Trial on BoNT for Androgenetic Alopecia Li et al., 2024 [31]	Randomized controlled trial	29	AGA	OnabotulinumtoxinA (BOTOX^®^, Allergan, Irvine, CA, USA)	Total dose: 100 U, 16 intradermal or intramuscular injection sites on half the scalp	Intradermal injections Hair density (hairs/cm^2^): Baseline—120.5 12 weeks—126.2 *p* = 0.217 Intramuscular injections Hair density (hairs/cm^2^): Baseline—118.3 12 weeks—136.1 *p* < 0.001	Alopecia areata: 1/29 (3.4%)
A study of combination unilateral subcutaneous botulinum toxin a treatment for androgenetic alopecia Tian et al., 2022 [32]	Randomized controlled trial	37	AGA	OnabotulinumtoxinA (BOTOX^®^, Allergan, Irvine, CA, USA)	Total dose: 100 U, intradermal injections on half the scalp	Hair density assessment Patients with increased hair density at 6 months BoNT group: 29.31% Placebo (NS): 12.88%	No AEs reported
Effectiveness and Safety of Botulinum Toxin Type A in the Treatment of Androgenetic Alopecia Zhou et al., 2020 [33]	Randomized clinical trial	63	AGA	OnabotulinumtoxinA (BOTOX^®^, Allergan, Irvine, CA, USA)	Total dose: 100 U, 30 intradermal injections on entire scalp 33 patients used concomitant 1 mg finasteride	Hair density assessment-BoNT alone Hair density (hairs/cm^2^) Baseline: 180.57 6 months: 208.04 12 months: 218.26 *p* < 0.001, comparison between baseline and 12 months Hair density assessment-BoNT + finasteride Hair density (hairs/cm^2^) Baseline: 178.21 6 months: 220.44 12 months: 234.01 *p* = 0.035, comparison between BoNT and BoNT + finasteride groups at 12 months (all other time points *p* > 0.05)	Headache: 1/63 (1.6%) Injection site pain: 3/63 (4.8%) Nausea: 1/63 (1.6%)
Efficacy of type A botulinum toxin treatment for androgenetic alopecia using ultrasound combined with trichoscopy Li et al., 2024 [34]	Randomized clinical trial	90	AGA	BoNT-A (Lanzhou Institute of Biological Products Co., Ltd., Qilihe District, Lanzhou City, China)	Total dose: 100 U, 20 intradermal injection sites on vertex and frontotemporal scalp	Hair density assessment Hair count (hairs/1 cm^2^ pre-defined area) Baseline—11 1 month—11 3 months—11	No AEs reported
Assessment of efficacy of different botulinum toxin A concentrations in the treatment of androgenetic alopecia assessed by dermoscopy Seoudy et al., 2024 [35]	Randomized controlled trial	32	AGA	BoNT-A (unspecified)	Total dose: 100 U, 15 intradermal injection sites on half the scalp	Dermoscopy assessment Patients with hair shaft diversity, yellow dots, vellus hairs and/or peripilar sign Baseline—6/32 (18.8%) 3 months—2/32 (6.2%) 6 months—2/32 (6.2%) *p* < 0.001, between baseline and 6 months Clinical assessment: Ludwig scale Baseline—4/27 (14.8%) Grade 1; 6/27 (22.2%) Grade 2; 17/27 (63%) Grade 3 3 months—20/27 (74.1%) Grade 1; 7/27 (25.9%) Grade 2; 0.27 (0%) Grade 3 6 months—19/27 (70.4%) Grade 1; 7/27 (25.9%) Grade 2; 1/27 (3.7%) Grade 3 *p* = 0.009, comparison between 6 months and baseline Clinical assessment: Norwood Hamilton scale Baseline- 0/5 (0%) Grade 2; 2/5 (40%) Grade 3; 3/5 (60%) Grade 4 3 months- 7/5 (80%) Grade 2; 1/5 (20%) Grade 3; 0/5 (0%) Grade 4 6 months- 3/5 (60%) Grade 2; 2/5 (40%) Grade 3; 0/5 (0%) Grade 4 *p* < 0.001, comparison between 6 months and baseline	Scalp irritation: 4/32 (12.5%) Headache: 10/32 (31.2%) Injection site pain: 1/32 (3.1%) Nausea: 1/32 (3.1%)
Efficacy and safety of botulinum toxin A in the treatment of female pattern hair loss Hu et al., 2024 [36]	Clinical trial	10	AGA	BoNT-A (unspecified)	Total dose: 100 U, 20 intradermal injection sites on half the scalp	Clinical improvement assessment Change in severity after 3 months Deterioration: 1/10 (10%) No change: 6/10 (60%) Mild improvement: 3/10 (30%)	No AEs reported
Assessing the efficacy and quality of Life improvements of botulinum toxin type a with topical minoxidil versus topical minoxidil in male androgenetic alopecia: a randomized controlled trial Yu et al., 2024 [37]	Randomized controlled trial	60	AGA	BoNT-A (unspecified)	Total dose: 100 U, 30 intradermal and intramuscular injection sites on entire scalp Used with daily 5% topical minoxidil	Clinical improvement assessment Average improvement (0 = no change, 3 = marked improvement) 2 months-post treatment: 0.96 4 months-post treatment: 2.06 *p* < 0.001, comparison between BTX group and placebo (5% minoxidil only) at 4 months	No AEs reported
Cephalalgia alopecia or nummular headache with trophic changes? A new case with prolonged follow-up Irimia et al., 2013 [38]	Case report	1	CA	BoNT-A (unspecified)	Total dose: 100 U, intramuscular injections surrounding alopecic patch	Subjective improvement assessment Baseline: 3 cm diameter alopecic patch 3 months post-treatment: complete resolution of patch	No AEs reported
Cephalalgic alopecia areata: a syndrome of neuralgiform head pain and hair loss responsive to botulinum A toxin injection Cutrer et al., 2006 [39]	Case report	1	CA	BoNT-A (unspecified)	Total dose: 100 U, intramuscular injections surrounding alopecic patch	Subjective improvement assessment Complete remission of head pain for 60 days and hair regrowth after 2nd BoNT session	No AEs reported
50 U BoNT							
Hair-to-Hair Trichoscopy: An Objective Method to Assess Effectiveness of Botulinum Toxin in a Clinical Trial for Androgenetic Alopecia Melo et al., 2023 [40]	Randomized clinical trial	13	AGA	OnabotulinumtoxinA (BOTOX^®^, Allergan, Irvine, CA, USA)	Total dose: 50 U, 10 intradermal or intramuscular injection sites on one 2 × 4 cm area on vertex and frontotemporal scalp	Tricholab^®^ H2H-matching assessment Average hair thickness Baseline—0.056 mm 24 weeks post-treatment: 0.057 mm Number of vellus hairs Baseline—152 24 weeks post-treatment—148 Number of terminal hairs Baseline—70 24 weeks post-treatment—82	No AEs reported
Efficacy of botulinum toxin A injection in the treatment of androgenic alopecia: A Comparative Controlled Study Nassar et al., 2022 [41]	Randomized controlled trial	62	AGA	BoNT-A (unspecified)	Total dose: 50 U, 30 intramuscular injection sites on entire scalp	Dermoscopy assessment Patients with hair shaft diversity Baseline—62/62 (100%) 6 months—50/62 (80.6%) *p* < 0.001, between baseline and 6 months Clinical assessment: Ludwig scale Baseline—12% Grade 1; 36% Grade 2; 17/27 52% Grade 3 6 months—36% Grade 1; 44% Grade 2; 0% Grade 3 Clinical assessment: Norwood Hamilton scale Baseline—50% Grade 3; 16.7% Grade 4, 33.3% Grade 5 6 months—16.7% Grade 1; 66.7% Grade 2; 16.7% Grade 3	No AEs reported
A small dose of botulinum toxin A is effective for treating androgenetic alopecia in Chinese patients Zhang et al., 2019 [42]	Prospective interventional study	24	AGA	BoNT-A (unspecified)	Total dose: 50 U, 30 intradermal injection sites on entire scalp	Clinical improvement assessment 3 months post-treatment: Obvious hair regrowth—9/24 (37.5%) No apparent hair regrowth—10/24 (41.6%) Progressing hair loss—5/24 (20.8%) 6 months post-treatment: Obvious hair regrowth—11/24 (45.8%) No apparent hair regrowth—8/24 (33.3%) Progressing hair loss—5/24 (20.8%)	No AEs reported
Efficacy of botulinum toxin in male androgenetic alopecia: A triple-blind, randomized clinical trial Melo et al., 2024 [43]	Randomized controlled trial	13	AGA	BoNT-A (unspecified)	Total dose: 50 U, 10 intradermal or intramuscular injection sites on one 2 × 4 cm area on vertex and frontotemporal scalp	Hair density assessment-vertex Hair density (hairs/cm^2^) Baseline—216.9 24 weeks—217.7 *p* = 0.408 Hair density assessment-frontotemporal Hair density (hairs/cm^2^) Baseline—216.0 24 weeks—218.8 *p* = 0.290	No AEs reported
45 U BoNT							
Amelioration of trichotillomania with onabotulinumtoxinA for chronic migraine Engel et al., 2023 [44]	Case report	1	TTM	BoNT-A (unspecified)	Total dose: 45 U, intradermal injections diffusely throughout scalp	Patient-reported improvement 12 weeks-post treatment: reports no urges to pull hair Subjective clinical assessment 12 weeks-post treatment: mild hair regrowth 1 year-post continual treatment: significant hair regrowth	No AEs reported
30 U BoNT							
The combination of platelet-rich plasma with botulinum toxin A in the treatment of hyaluronic acid embolic cutaneous necrosis and alopecia Guo et al., 2022 [45]	Case report	1	Filler-induced alopecia (due to intravascular injection causing scalp necrosis)	BoNT-A (unspecified)	Total dose: 30 U, intradermal injections in alopecic patch Performed with PRP injections	Subjective clinical assessment 87 days after treatment: hair regrowth except for a 2 × 2 cm permanent cicatricial alopecia	No AEs reported
BoNT dosage not specified							
Botulinum toxin treatment of cephalalgia alopecia increases substance P and calcitonin gene-related peptide-containing cutaneous nerves in scalp Cutrer et al., 2010 [39]	Case report	1	CA	BoNT-A (unspecified)	Total dose: not specified, intramuscular injections surrounding alopecic patch	Subjective improvement assessment Remission of scalp pain for about 6 weeks, with regrowth of hair. Subsequent treatments induced remissions lasting 4–12 weeks	No AEs reported

AA, alopecia areata; AE, adverse event; AGA, androgenetic alopecia; BoNT, botulinum toxin; NS, normal saline; PRP, platelet-rich-plasma; TE, telogen effluvium; TTM, trichotillomania. Underlined text = measurement method of BoNT efficacy.

**Table 2:** Studies describing patients who received BoNT injections in the scalp for the treatment of craniofacial hyperhidrosis, organized in order of descending injection dose.

Title, Author and Date	Study Type	# of Patients	Hair/Scalp Condition	BoNT Type (Formulation)	Dose	Results	Adverse Events (AEs)
2250 U BoNT							
Postmenopausal craniofacial hyperhidrosis treated with botulinum toxin type B Cabreus 2019 [46]	Randomized controlled trial	8	HH	BoNT type B (NeuroBloc^®^; Eisai Europe, Hatfield, UK)	Total dose: 2250 U, injected every 15 mm across the frontal, temporal, and occipital scalp, forehead, glabella, intraocular, and perioral areas	DLQI Assessment Placebo: 90% improvement from baseline BoNT-B group: 18% decline from baseline HDSS Assessment Placebo average: 2.8 BoNT-B average: 1 Gravimetric Data (sweat rate) Assessment Placebo average: 0.035 mg/min BoNT-B average: 0.0012 mg/min	Forehead stiffness: 2/3 (66.7%) Nausea during injection: 1/3 (33.3%) Increased sweating from back: 1/3 (33.3%) Dry mouth: 1/3 (33.3%)
250 U BoNT							
Botulinum toxin B in the treatment of craniofacial hyperhidrosis Karlqvist 2014 [47]	Clinical trial	38	HH	RimabotulinumtoxinB (NeuroBloc^®^; Eisai Co., Ltd., Tokyo, Japan)	Total dose: 250 U, injected every 15 mm across the frontal and occipital scalp	DLQI assessment Pre-treatment: 13.1 ± 1 Post-treatment: 5 ± 1 *p* < 0.001 Trans-epidermal water loss assessment Pre-treatment: 52 ± 31 g/m^2^/h Post-treatment: 18 ± 7 g/m^2^/h *p* < 0.001 Gravimetric data (sweat rate) assessment Pre-treatment: 0.07 ± 0.08 mg/min Post-treatment: 0.02 ± 0.05 mg/min *p* < 0.05 Clinical improvement assessment Significant-complete reduction in sweating: 33/38 (87%) Moderate reduction in sweating: 4/38 (10%)	Forehead stiffness: 18% Eyebrow drooping: 18% Compensatory sweating: 11% Local skin dryness: 5% Dryness of the mouth: 3% Local bruising: 3% Worsening of migraine: 3%
A unique case of primary focal hyperhidrosis and treatment Hannan 2022 [48]	Case report	1	HH	AbobotulinumtoxinA (Dysport^®^; Galderma, Dallas, TX, USA)	Total dose: 150 U, injected evenly into affected areas	Clinical improvement assessment At the 3-month follow-up visit, patient reported decreased gustatory sweating	No AEs reported
100 U BoNT							
Botulinum Toxin for the Treatment of Postmenopausal Craniofacial Hyperhidrosis Patrick 2024 [49]	Case report	1	Craniofacial hyperhidrosis (HH)	BoNT-A (unspecified)	Total dose: 100 U, injected along the superior forehead and occipital hairline	Clinical improvement assessment Patient reported significant improvement in sweating and returned for four follow-up BTX sessions that were 11 months apart	No AEs reported
Effect of Botulinum Toxin in Stellate Ganglion for Craniofacial Hyperhidrosis: a Case Report Park 2021 [50]	Case report	1	HH	OnabotulinumtoxinA (BOTOX^®^; Allergan, Irvine, CA, USA)	Total dose: 100 U, injected into bilateral stellate ganglion	Clinical improvement assessment Sweating reduced at 6-month follow-up	No AEs reported
Postmenopausal craniofacial hyperhidrosis Eustace 2018 [51]	Case series	11	HH	BoNT-A (unspecified)	Total dose: 100 U, injected into hairline and nape of neck	Clinical improvement assessment 64% (7/11) patients noticed complete remission of symptoms and 36% (4/11) had no improvement	No AEs reported
Hyperhidrosis of Face and Scalp: Repeated Successful Treatment with Botulinum Toxin Type A Komericki 2012 [52]	Case report	1	HH	OnabotulinumtoxinA (BOTOX™; Allergan, Irvine, CA, USA)	Total dose: 100 U, 30 injection points across the upper forehead and scalp	Clinical improvement assessment Sweating completely stopped after treatments	No AEs reported
BoNT dosage not specified							
Craniofacial Hyperhidrosis in Post-Menopausal Women Alsharqi 2012 [53]	Case series	2	HH	BoNT-A (unspecified)	Total dose: not specified, BoNT-A injected into hyperhidrotic areas on face and scalp	Clinical improvement assessment “Patient showed good response”	No AEs reported

AE, adverse event; BoNT, botulinum toxin; Dermatology Life Quality Index, DLQI; HDSS, Hyperhidrosis disease severity score. Underlined text = measurement method of BoNT efficacy.

**Table 3:** Studies describing patients who received BoNT injections in the scalp for the treatment of other scalp conditions, organized in order of descending injection dose.

Title, Author and Date	Study Type	# of Patients	Hair/scalp Condition	BoNT Type (Formulation)	Dose	Results	Adverse Events (AEs)
200 U BoNT							
Botulinum Toxin Type A- Treatment of a Patient with Multiple Cutaneous Piloleiomyomas Sifaki et al., 2008 [54]	Case report	1	Scalp pain (etiology: cutaneous piloleiomyomas)	BoNT-A (unspecified)	Total dose: 200 U, 10–20 intralesional injections	Symptom severity assessment Patient-reported in a 0–10 range (0 = no pain, 10 = most severe pain) Baseline: 10/10 4 days post-BoNT: 2/10	No AEs reported
150 U BoNT							
Evaluation of the effect of botulinum toxin injection in aggravating or improving seborrheic dermatitis symptoms: A prospective, single-arm clinical trial Bazargan et al., 2023 [55]	Clinical trial	20	Seborrheic dermatitis	BoNT-A (MASPORT^®^, MasoonDarou Pharmaceutical Company, Karaj, Alborz Province, Iran)	Total dose: 150 U, intradermal injections in the scalp hairline	Extent of scalp involvement Rated on a scale 1–5 (1 = less than 10%, 2 = 11–30%, 3 = 31–50%, 4: 51–70%, 5: more than 70%) Baseline: 2.10 ± 1.02 1 month-post BTX-A: 1.15 ± 1.23 (*p* > 0.05) Seborrheic dermatitis severity Patient-reported symptom severity on a scale 0–3 (0 = symptom not present, 3 = symptom is at most severe condition) Baseline: Skin erythema: 0.25 ± 0.44 Skin sebum: 1.35 ± 1.04 Scaling: 1.60 ± 0.68 1 month-post BTX-A: Skin erythema: 0.15 ± 0.37 Skin sebum: 1.25 ± 1.16 Scaling: 1.40 ± 0.68 (*p* = 0.528, overall severity score compared to baseline)	No AEs reported
Folliculitis Responds to Botulinum Toxin: Is It Possible? Tamura et al., 2007 [56]	Case series	4	Folliculitis decalvans (biopsy-confirmed)	OnabotulinumtoxinA (BOTOX^®^, Allergan, Irvine, CA, USA)	Total dose: 60–150 U, 2.5 U injected in intralesional and perilesional locations	Patient 1: 80 U BoNT-A total Reduced secretion 30 days after injection Signs of hair growth 30 days after injection Patient 2: 60 U BoNT-A total Reduced secretion Discrete, diffuse, and slow hair growth Patient 3: 150 U BoNT-A total Reduced secretion 30 days after injection Signs of hair growth 30 days after injection Patient 4: 100 U BoNT-A total Reduced secretion 6 months after injection. No significant hair growth	No AEs reported
100 U BoNT							
Botulinum toxin A as an alternative treatment for folliculitis decalvans Neri et al., 2023 [57]	Case report	1	Folliculitis decalvans (biopsy-confirmed)	BoNT-A (unspecified)	Total dose: 100 U, intralesional injections	Clinical assessment Improvement in inflammatory lesions and elimination of scalp itch within “a few days” Full remission of the disorder within 4 months of first BoNT-A session Condition remained stable with no relapses for the following 5-year follow up period	No AEs reported
Therapeutic effect of botulinum toxin A on folliculitis dissecans of the scalp Neri et al., 2024 [58]	Case report	1	Dissecting folliculitis of the scalp (biopsy-confirmed)	OnabotulinumtoxinA (BOTOX^®^, Allergan, Irvine, CA, USA)	Total dose: 100 U, injected intradermally outlining the infection sites	2 weeks post-BoNT: resolution of scalp pain 8 weeks post-BoNT: resolution of abscesses and swelling with significant increase in hair density over infection sites	No AEs reported
Successful Treatment of Refractory Trichodynia With Onabotulinumtoxin-A Alhomida et al., 2024 [22]	Case report	1	Trichodynia (unknown etiology)	OnabotulinumtoxinA (BOTOX^®^, Allergan, Irvine, CA, USA)	Total dose: 100 U, intradermal injections throughout the scalp	Symptom severity assessment Patient-reported in a 0–10 range (0 = no pain, 10 = most severe pain) Baseline: 10/10 One-month post-BoNT: 0/10 Six-weeks post-BoNT: 6/10 Three-months post-BoNT: 10/10	No AEs reported
25–50 U BoNT							
Intradermal Botulinum Toxin A Injection for Scalp Sebum Secretion Regulation: A Multicenter, Randomized, Double-Blinded, Placebo-Controlled, Prospective Study in Chinese Subjects Li et al., 2023 [59]	Randomized controlled trial	49	Scalp hyperseborrhea	Chinese BoNT-A (Hengli^®^, Lanzhou Institute of Biology, Lanzhou, China)	Total dose: 50–65 U, 25 intradermal injection sites throughout the scalp	Intradermal BoNT-A treatment significantly reduced the scalp sebum secretion at 24-, 48-, and 72-h post-shampooing at the 1- and 3-month follow-up (*p* < 0.05)	scalp tightness dizziness insomnia itchiness folliculitis hematoma tinnitus (% of patients affected not specified)
Parry-Romberg Syndrome Vasculopathy and Its Treatment with Botulinum Toxin Borodic et al., 2014 [60]	Case report	1	Linear scleroderma/En coup de sabre morphea	BoNT-A (unspecified)	Total dose: 50 U, 6 injections along the forehead and frontotemporal scalp	Clinical improvement assessment After first BoNT injection: “dramatic improvement in pain” After second BoNT injection: “dramatic relief of pain, hair loss, and improvement of memory”	No AEs reported
Botulinum Toxin for Scalp Dysthesia Phan et al., 2022 [61]	Case report	1	Scalp dysthesia (unknown etiology)	BoNT-A (unspecified)	Total dose: 40 U, 20 intradermal injections across half the scalp	Symptom severity assessment Patient-reported in a 0–10 range (0 = no pain, 10 = most severe pain) Baseline: 4/10 8 weeks post-BoNT: 2/10 16 weeks post-BoNT: 4/10	No AEs reported
Improvement of “En Coup de Sabre” Morphea and Associated Headaches with Botulinum Toxin Injections Rimoin et al., 2016 [62]	Case report	1	Linear scleroderma/En coup de sabre morphea	BoNT-A (unspecified)	Total dose: 25 U, 10 intralesional and perilesional injections	Cosmetic appearance assessment Physician-reported based on pigmentation and sclerotic changes in a 0–10 range (1 = least severe, 10 = most severe) Baseline: 10/10 4 months post-BoNT: 4/10 6 months post-BoNT: 4/10 9 months post-BoNT: 3/10 12 months post-BoNT: 3/10	No AEs reported

AA, alopecia areata; AE, adverse event; AGA, androgenetic alopecia; BoNT, botulinum toxin; NS, normal saline; PRP, platelet-rich-plasma; TE, telogen effluvium; TTM, trichotillomania; VAS, visual analogue scale. Underlined text= measurement method of BoNT efficacy.

## Data Availability

No new data were created or analyzed in this study.
